# Continuous and Intermittent Alcohol Free-Choice from Pre-gestational Time to Lactation: Focus on Drinking Trajectories and Maternal Behavior

**DOI:** 10.3389/fnbeh.2016.00031

**Published:** 2016-03-03

**Authors:** Anna Brancato, Fulvio Plescia, Gianluca Lavanco, Angela Cavallaro, Carla Cannizzaro

**Affiliations:** ^1^Department of Sciences for Health Promotion and Mother and Child Care “Giuseppe D'Alessandro”, University of PalermoPalermo, Italy; ^2^Department BioNeC, University of PalermoPalermo, Italy

**Keywords:** female rats, two-bottle choice, drinking trajectories, pregnancy, lactation, maternal behavior

## Abstract

**Background:** Alcohol consumption during pregnancy and lactation induces detrimental consequences, that are not limited to the direct in utero effects of the drug on fetuses, but extend to maternal care. However, the occurrence and severity of alcohol toxicity are related to the drinking pattern and the time of exposure. The present study investigated in female rats long-term alcohol drinking trajectories, by a continuous and intermittent free-choice paradigm, during pre-gestational time, pregnancy, and lactation; moreover, the consequences of long-term alcohol consumption on the response to natural reward and maternal behavior were evaluated.

**Methods:** Virgin female rats were exposed to home-cage two-bottle continuous- or intermittent “alcohol (20% v/v) vs. water” choice regimen along 12 weeks and throughout pregnancy and lactation. Animals were tested for saccharin preference, and maternal behavior was assessed by recording dams' undisturbed spontaneous home-cage behavior in the presence of their offspring.

**Results:** Our results show that the intermittent alcohol drinking-pattern induced an escalation in alcohol intake during pre-gestational time and lactation more than the continuous access, while a reduction in alcohol consumption was observed during pregnancy, contrarily to the drinking trajectories of the continuous access-exposed rats. Long-term voluntary alcohol intake induced a decreased saccharin preference in virgin female rats and a significant reduction in maternal care, with respect to control dams, although the intermittent drinking produced a greater impairment than the continuous-access paradigm.

**Conclusion:** The present data indicate that both alcohol-drinking patterns are associated to modifications in the drinking trajectories of female rats, in pre-gestational time, during pregnancy and lactation. Moreover, long-lasting alcohol intake can affect sensitivity to natural rewarding stimuli and maternal behavior and sensitivity to natural rewarding stimuli in a pattern–related manner. This study underlies the importance of modeling human alcohol habit and its consequences on the mother-infant dyad, in order to prevent detrimental effects on offspring development and maturation.

## Introduction

Recent official reports have described a marked increase in binge (heavy episodic) drinking among young women in Europe (Sassi, 2015). These reports have outlined the potential impact of binge-drinking behavior from a health, economic, and social perspective, but no mention has been made on the potential teratogenic effect of alcohol. Despite, the fact that most women in the 16–24 year-old age group reporting binge drinking once a week are not actually pregnant, the majority of pregnancies are unplanned. Therefore women often continue alcohol drinking into the early weeks of an unplanned pregnancy, a period in which the fetus is particularly vulnerable to alcohol toxicity. In this regard, a recent international cross-cohort study shows high prevalence of alcohol use during pregnancy and high levels of binge drinking (O'Keeffe et al., 2015).

The detrimental consequences exerted by gestational alcohol intake are not limited to the direct in utero effects of the drug on fetuses, but extend to maternal care, which contributes significantly to offspring's development and maturation. Moreover, alcohol ingestion during pregnancy is likely to continue during the breastfeeding period (O'Keeffe et al., 2015), exacerbating alcohol consequences on both the mother and the newborn. Indeed, the dam-infant interaction is a fundamental relationship, functionally necessary to the infant's wellness and to the development of his own behavioral repertoire (Gubernick and Alberts, 1983; Kinsley, 1994; Jacobson, 1997; Fleming et al., 1999; Gonzalez et al., 2001; Stamps, 2003). Research on the animal model has contributed to the analysis of maternal behavior in a controlled environment. Focusing on the rat, dams build a nest, retrieve, and gather pups in this habitat, lick the anogenital region of the infants to stimulate defecation and micturition and also adopt a crouching posture over the progeny to facilitate nipple attachment and suckling (Blass, 1990). Disruptions of this behavioral repertoire modify developmental processes, that affect various behavioral, physiological, and morphological traits of the progeny (Kinsley, 1994; Rosenblatt and Snowdon, 1996; Roth et al., 2004). To our knowledge, few studies have reported on alterations in maternal care induced by gestational alcohol intake and they were limited to alcohol administration to dams during pregnancy and/or after parturition (Pepino et al., 2002; McMurray et al., 2008; Pueta et al., 2008). Narcoleptic doses of ethanol to the dams dramatically disrupt prolactin and oxytocin release in dams during pregnancy and/or after parturition, as well as parental care. Notably, a sub-narcoleptic dose of ethanol administered to dams during the first postnatal week is able to disrupt fixed action patterns relevant to maternal care (Pepino et al., 2002; Pueta et al., 2008). Generally, in the population, alcohol use during pregnancy derives from long-lasting habit to voluntary alcohol drinking, and therefore alcohol administration to the dams, whatever the dosage, is quite far from a translational perspective. Rather, a commonly used approach for modeling human alcohol consumption in rodents is the alcohol preference study, in which animals are given a choice between water and alcohol solutions of various strength, and the amount of each fluid consumed is measured. Indeed, the free-choice paradigm provides a controlled environment in which the animals voluntarily drink alcohol, according to their internal milieu or the environmental cues. Recently, a growing body of literature indicates that long-term training in the intermittent alcohol free-access procedure leads to binge-like drinking episodes generating increased alcohol preference and high blood alcohol concentrations in rats (Wise, 1973; Loi et al., 2010; Carnicella et al., 2014; Peris et al., 2015). As in humans, the drinking pattern represents a determining factor for the development of specific neuronal and behavioral adaptations (Stuber et al., 2008; Hopf et al., 2010) that may also discretely influence reward processing and gestational and postnatal events. Given these preliminary remarks, in the present study, we investigated long-term alcohol intake by a continuous and intermittent free-choice paradigm from pre-gestational - to pre-weaning time, in order to measure alcohol drinking trajectories in different physiological conditions such as virginity, pregnancy, and lactation. As pups generally represent an important hedonic source for the mother (Kehoe and Shoemaker, 1991), we hypothesized, that long-term alcohol consumption could alter the reward processing in female rats. Thus, the consequences of the two drinking patterns on the response to natural rewards, and on post-partum maternal behavior were investigated. Since, gestational alcohol treatment alone has not been shown to affect the onset of maternal behavior, opposite to post-partum ethanol exposure, it appears that the duration and timing of exposure play a crucial role in the occurrence of alcohol toxicity.

The outcomes of this study will help to provide a wider framework of the interactions between alcohol intake and maternal care, that takes into consideration long-term habit to two different drinking patterns throughout significant stages of female life, thus giving the present research a translational heuristic value.

## Materials and methods

### Pre-gestational assessments

#### Animals and housing conditions

Adult female Wistar rats (Harlan, Udine, Italy; 8 weeks, 200–220 g) were housed individually in standard rat cages (40 cm × 60 cm, 20 cm in height) and maintained in a temperature- (22 ± 2°C) and humidity- (55 ± 5 %) controlled room, with *ad libitum* access to water and food. The colony was maintained on 12 h light/dark cycle (08:00 to 20:00) and rats were gently handled for 3 min per day for a week before the experimental procedures. All the experiments were conducted in accordance with the regulations of the Committee for the Protection and Use of Animals of the University of Palermo, in accordance with the current Italian legislation on animal experimentation (D.L. 116/92) and the European directives (2010/63/EU).

#### Two-bottle “20% alcohol vs. water” choice drinking paradigms

Female rats were matched for body weight, and randomly assigned to one of the three experimental groups (*n* = 24): continuous alcohol access- rats (CAR, *n* = 24)—20% v/v alcohol with continuous access (24 h/day, 7 days/7week); intermittent alcohol access-rats (IAR, *n* = 24)—20% v/v alcohol with intermittent access (three 24 h drinking sessions per week on Monday, Wednesday, and Friday) and 2 water bottles for the remaining 4 days; water-drinking controls (CTR, *n* = 24)—receiving two bottles of tap water.

Alcoholic solution (20 % v/v) was daily prepared by diluting ethanol 96° (Carlo Erba Reagenti, Italy) with tap water. Bottles (Tecniplast, Italy) were refilled every day with fresh solution, and presented every day at lights off, in alternative left-right position, to avoid side preference. Alcohol- and water intake were carefully measured by weighing the bottles before delivery, after 1 h and every 24 h (0.1 g accuracy); possible fluid spillage was calculated and subtracted before data analysis according to Loi et al. (2014). Data analyzed in the present study refer only to alcohol intake during the drinking sessions of the intermittent-access groups.

#### Saccharin preference test

This test is validated as index of anhedonia (Pucilowski et al., 1993). It measures rat's sensitivity toward natural rewarding stimuli, and is based on the rewarding properties of saccharin or sucrose. At week 12, at the end of the last drinking session, 8 cages from each experimental group were supplied with two identical drinking bottles filled with water (habituation phase). On the test day, at 24 h alcohol deprivation for both groups, one bottle of water was replaced by a bottle containing a solution with 0.2% (w/v) saccharin (Sigma Aldrich, Italy), and rats were offered to freely choice between the two options over a 24 h period. The location of the two bottles was randomly alternated to avoid location preference bias. The total volume of fluid intake was recorded, and the preference ratio was calculated by measuring the volume of the saccharin solution consumed with respect to total fluid intake.

### Gestational and postnatal assessments

#### Animals and housing conditions

Twelve female rats from each experimental group were randomly selected and housed, with a single breeder male. The day when the pregnancy was confirmed, designated as gestational day 1 (GD 1), eight female rats from each experimental group were housed in standard maternity cages, filled with wood shavings. Ethanol consumption was not measured on day 1 and 2, when the male was present in the cage (Allan et al., 2003). Females continued to drink throughout pregnancy, accordingly to the respective two-bottle choice paradigm. Dams were inspected twice daily for delivery, and the day of parturition was considered as postnatal day 0 (PND 0); dams and litters were kept in a nursery, under proper temperature-controlled conditions (24 ± 2°C).

#### Two-bottle “20% alcohol vs. water” choice drinking paradigms

During gestation and lactation, female rats were exposed to the two-bottle choice paradigm at the same pre-gestational experimental conditions. In particular, alcohol intake was recorded from GD 1 and continued throughout lactation until PND 21. Dams' body weight was measured two times a week during gestational and post-partum time, in order to minimize animal distress.

#### Maternal behavior assessment

Maternal behavior was assessed by a silent and unseen observer who recorded dams' spontaneous behavior in the presence of the offspring in the home cage, under undisturbed conditions, through direct periodic observations along 15 days from PND 1 to PND 21 (from Monday to Friday). The assessment was performed at 9:00, 11:30 am, 01:30, 03:00 pm, and consisted of 3 trials of 20 s-observation, for a total of 12 instantaneous observations per animal per day (3 observations × 4 times per day × 15 days = 180 observations/dam). The 20 sec time observation allowed an exact identification of the ongoing behavior. The behavioral parameters scored were retrieval, nursing (arched-back, blanket, passive), pup care (licking, anogenital licking), dam self-care (self-grooming, eating, drinking), and others (rearing, moving, resting, standing out of nest). A detailed description of the behavioral categories can be found in Capone et al. (2005). The observations were carried out during diurnal time, when animals behave more maternally (Grota and Ader, 1969, 1974). Original data were recorded using dichotomous scores during the instantaneous sampling (0/1). In particular, score 0 was assigned when the behavior was not shown in the interval of observation, while score 1 was assigned when the behavior was performed. The original dichotomous scores (0/1) were transformed into quantitative variables, adding the original scores of the instantaneous samples within each time point, ranging from 0 (behavior never shown) to 3, (the maximum number of instantaneous samples within the time point), and along the 4 time points. Thus, a daily score ranged between 0 and 12.

In order to get a general framework of the behavioral measurements, a daily index of overall maternal behavior (MB-I) was calculated as follows: (maternal score) − (non-maternal score)/(maternal score) + (non-maternal score). The index ranges from −1 (totally non-maternal behaviors) to +1 (totally maternal behaviors).

### Statistical analysis

#### Two-bottle “20% alcohol vs. water” choice drinking paradigms

Data on alcohol intake, expressed in g/kg/day, and total fluid intake (ml/kg) refer to the drinking sessions of both alcohol-drinking groups, and were analyzed by a two-way analysis of variance for repeated measures (RM two-way ANOVA), considering “alcohol drinking pattern” as the between subjects factor, and “time” (days, gestational, and lactation periods) as the repeated measures factor followed by a Bonferroni *post-hoc* test (α = 0.05). Data from the mean alcohol intake after 1 h, the total sum of alcohol intake over the drinking sessions and the total fluid consumption were analyzed by separate Student's *t-*test for two-tailed unpaired measures. Alcohol drinking behavior was also analyzed comparing maternal alcohol consumption during gestation and lactation to pre-gestational basal intake in both alcohol drinking groups by a one-way ANOVA for paired measures (*n* = 8) followed by a Bonferroni *post-hoc* test (α = 0.05). Data are reported as mean ± S.D. Statistical significance was set at *p* < 0.05.

#### Saccharin preference test

Results were analyzed by using one-way ANOVA. Bonferroni *post-hoc* test was employed, when necessary (α = 0.05). Data are reported as mean ± S.D. Statistical significance was set at *p* < 0.05.

#### Maternal behavior

Data on daily maternal behavior scores were analyzed by RM two-way ANOVA, considering “alcohol drinking pattern” or “treatment” as the between subject factor, and “time” as the repeated measures factor. MB-I scores were evaluated by using RM one-way ANOVA. Bonferroni *post-hoc* test was employed, when necessary (α = 0.05). Data are reported as mean ± S.D. Statistical significance was set at *p* < 0.05.

## Results

### Pre-gestational assessments

#### Alcohol intake along the two-bottle “20% alcohol vs. water” choice paradigms

**Figure 1:**
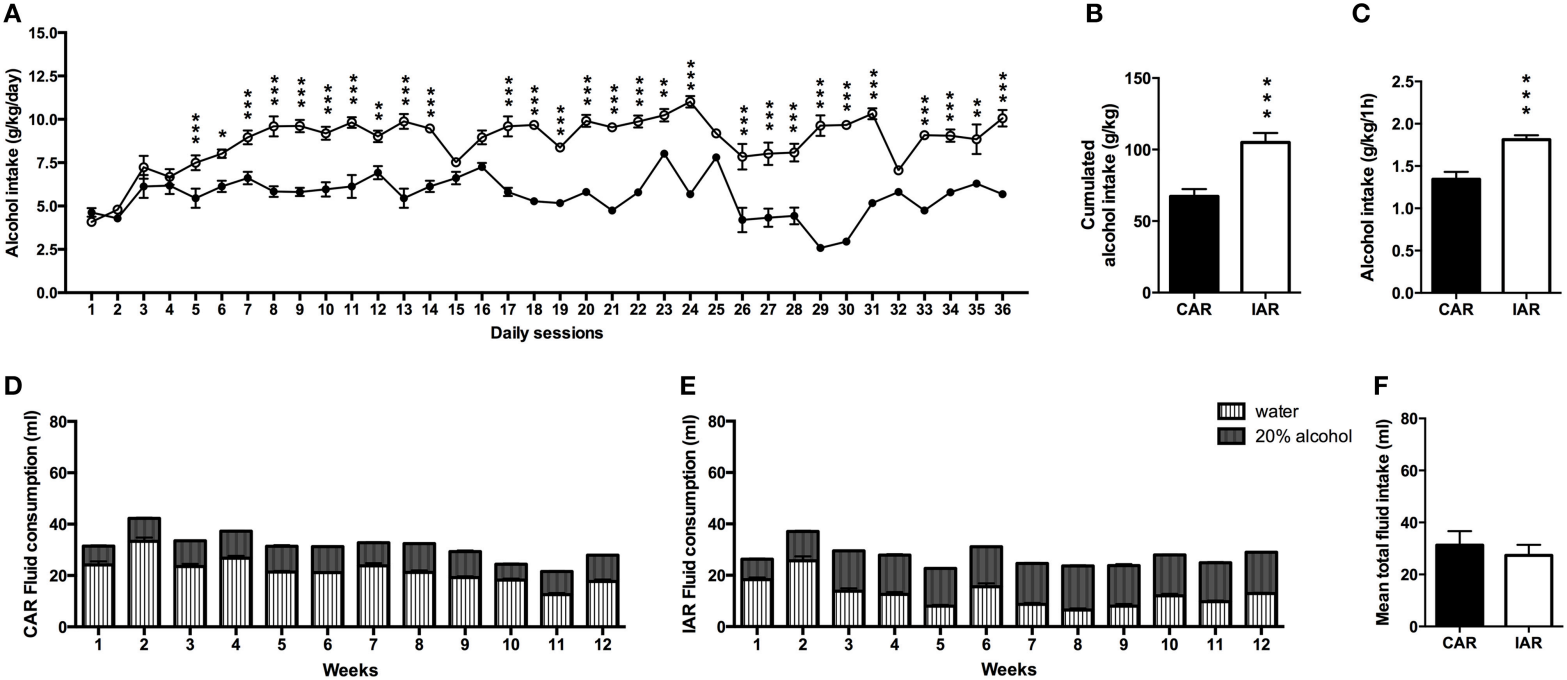
**Pre-gestational drinking behavior**. **(A)** Alcohol intake (expressed in g/kg/day) during the 36 pre-gestational drinking sessions in female rats exposed to the home cage, 2-bottle “alcohol vs. water” choice regimen, under the following conditions: CAR, 20% v/v alcohol with continuous access; IAR, 20% v/v alcohol with intermittent access. Results from Bonferroni *post-hoc* analysis showed a significantly higher alcohol intake in IAR, when compared to CAR, on days 5 (*t* = 16.125, *p* < 0.001), 6 (*t* = 5.188, *p* < 0.001), 7 (*t* = 6.427, *p* < 0.001), 8 (*t* = 8.561, *p* < 0.001), 9 (*t* = 12.09, *p* < 0.001), 10 (*t* = 8.868, *p* < 0.001), 11 (*t* = 10.11, *p* < 0.001), 12 (*t* = 5.746, *p* < 0.001), 13 (*t* = 12.14, *p* < 0.001), 14 (*t* = 9.174, *p* < 0.001), 16 (*t* = 4.671, *p* < 0.001), 17 (*t* = 10.37, *p* < 0.001), 18 (*t* = 12.05, *p* < 0.001), 19 (*t* = 8.776, *p* < 0.001), 20 (*t* = 11.24, *p* < 0.001), 21 (*t* = 13.16, *p* < 0.001), 22 (*t* = 11.21, *p* < 0.001), 23 (*t* = 6.084, *p* < 0.001), 24 (*t* = 14.62, *p* < 0.001), 25 (*t* = 3.794, *p* < 0.001), 26 (*t* = 11.51, *p* < 0.001), 27 (*t* = 20.13, *p* < 0.001), 28 (*t* = 9.102, *p* < 0.001), 29 (*t* = 19.12, *p* < 0.001), 30 (*t* = 18.44, *p* < 0.001), 31 (*t* = 14.12, *p* < 0.001), 32 (*t* = 3.410, *p* < 0.05), 33 (*t* = 11.88, *p* < 0.001), 34 (*t* = 8.977, *p* < 0.001), 35 (*t* = 6.714, *p* < 0.001), and 36 (*t* = 12.02, *p* < 0.001). **(B)** Total sum of alcohol consumed over the entire period. **(C)** Mean alcohol consumption following the first hour of access. **(D)** Total fluid intake (ml) in CAR over the 12 pre-gestational weeks. **(E)** Total fluid intake (ml) in IAR over the 12 pre-gestational weeks. **(F)** Mean fluid consumption over the entire period. Each point or bar is the mean ± SD of *n* = 24 rats. ^*^p < 0.05, ^**^p < 0.01, ^***^p < 0.001 vs. CAR.

Alcohol consumption expressed in g/kg/day was analyzed by RM two-way ANOVA, that indicated significant effect of days [*F*_(35, 1610)_ = 2728.89, *p* < 0.0001], alcohol drinking pattern [*F*_(1, 46)_ = 1106.49, *p* < 0.0001] and their interaction [*F*_(35, 1610)_ = 22.40, *p* < 0.0001]. Results from Bonferroni *post-hoc* analysis showed a significant increase in alcohol intake in IAR with respect to CAR as indicated in Figure 1A. A two-tailed Student *t-*test on the total amount of alcohol consumed over the 36 drinking sessions showed higher values in IAR (*t* = 13.56, *df* = 46, *p* < 0.001), with respect to CAR (Figure 1B). Moreover, after the first hour of alcohol exposure during the 36 drinking sessions, IAR displayed higher mean alcohol intake with respect to CAR (*t* = 3.139, *df* = 22, *p* = 0.0048; Figure 1C).

The analysis of the drinking behavior showed that CAR alcohol intake did not exceed the water one (Figure 1D); on the other hand, IAR consumed more alcohol than water, starting from the third week onwards (Figure 1E). Two-tailed Student's *t*-test on mean total fluid intake over the 12 pre-gestational weeks, indicated no significant differences between the two groups (*t* = 2.015, *df* = 22, *p* = 0.0653; Figure 1F).

#### Saccharin preference test

**Figure 2:**
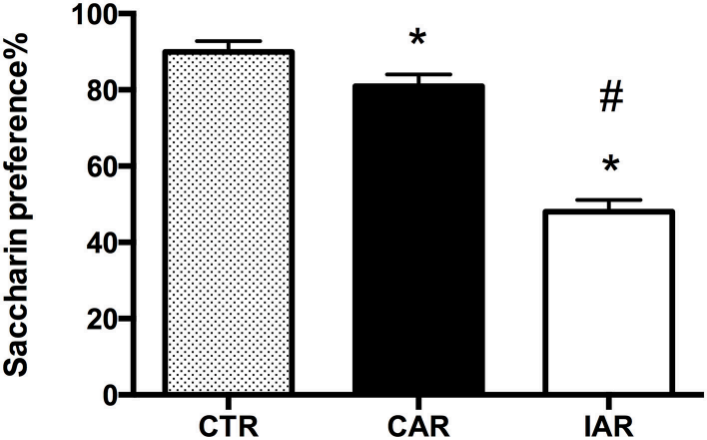
**Saccharin preference test in female rats exposed to the home cage, 2-bottle “alcohol vs. water” choice regimen, under the following conditions: CAR, 20% v/v alcohol with continuous access; IAR, 20% v/v alcohol with intermittent access**. Each value represents the mean ± SD. of 8 rats. ^*^p < 0.001 vs. CTR; ^#^*p* < 0.001 vs. CAR.

On the day of the experiment, at 24 h alcohol deprivation, animals were subjected to the saccharin preference test, in order to evaluate their response to a natural reward. Animals consumed the following amounts of saccharin solution: CTR 236.0 ± 42.8 g/kg; CAR 193.3 ± 24.4 g/kg; IAR 102.7 ± 29.7 g/kg. One-way ANOVA on saccharin preference showed significant differences among the three experimental groups [*F*_(2, 21)_ = 429.7, *p* < 0.001]. In details, Bonferroni *post-hoc* test highlighted a significant decrease in saccharin choice in both CAR (*t* = 5.992, *p* < 0.001) and IAR (*t* = 27.85, *p* < 0.001) with respect to CTR. Moreover, IAR displayed a significantly lower preference for saccharin compared to CAR (*t* = 21.86, *p* < 0.001; Figure 2).

### Gestational and postnatal assessments

#### Alcohol intake along the two-bottle “20% alcohol vs. water” choice paradigms

**Figure 3:**
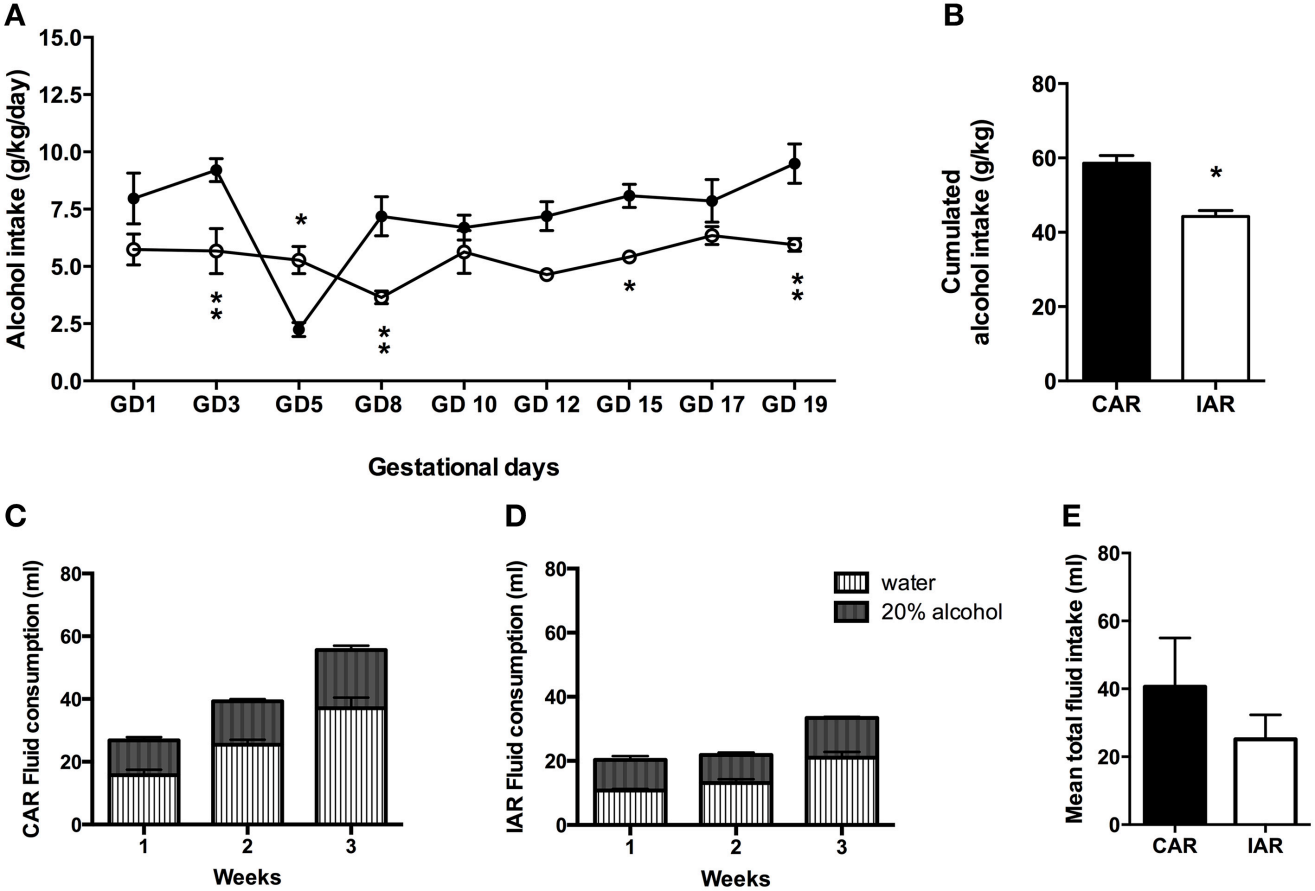
**Gestational drinking behavior**. **(A)** Alcohol intake (expressed in g/kg/day) during gestational days over 9 drinking sessions in female rats exposed to the home cage, 2-bottle “alcohol vs. water” choice regimen, under the following conditions: CAR, 20% v/v alcohol with continuous access; IAR, 20% v/v alcohol with intermittent access. Bonferroni *post-hoc* test indicated a significant decrease in IAR with respect to CAR on GD3 (*t* = 3.794, *p* < 0.01), GD5 (*t* = 3.252, *p* < 0.05), GD8 (*t* = 3.779, *p* < 0.01), GD15 (*t* = 2.856, *p* < 0.05), and GD19 (*t* = 3.799, *p* < 0.01). **(B)** Total sum of alcohol consumed over the entire period. **(C)** Total fluid intake (ml) in CAR over the 3 gestational weeks. **(D)** Total fluid intake (ml) in IAR over the 3 gestational weeks. **(E)** Mean fluid consumption over the entire period. Each point or bar is the mean ± S.E.M. of *n* = 8 rats. ^*^p < 0.05, ^**^p < 0.01 vs. CAR.

Daily alcohol intake in CAR and IAR during gestation is shown in Figure 3A. The RM Two-way ANOVA on g/kg/day of alcohol consumed by both groups revealed significant effect of gestation [*F*_(8, 112)_ = 8.03, *p* < 0.0001], alcohol drinking pattern [*F*_(1, 14)_ = 17.82, *p* = 0.0009], and their interaction [*F*_(8, 112)_ = 5.83, *p* < 0.0001]. In particular, Bonferroni *post-hoc* test indicated a significant decrease in IAR with respect to CAR, as reported in Figure 3A. A two tailed Student' *t*-test performed on the total amount of alcohol consumed over the 9 gestational drinking sessions showed that IAR reduced their alcohol intake during pregnancy (*t* = 5.497, *df* = 14, *p* < 0.001), when compared to CAR (Figure 3B). Total fluid intake over the 3 gestational weeks is reported in Figure 3C (CAR) and Figure 3D (IAR). Two-tailed Student's *t*-test on mean fluid intake over the 3 gestational weeks, indicated no significant differences between the two groups (*t* = 1.657, *df* = 4, *p* = 0.1728; Figure 3E).

**Figure 4:**
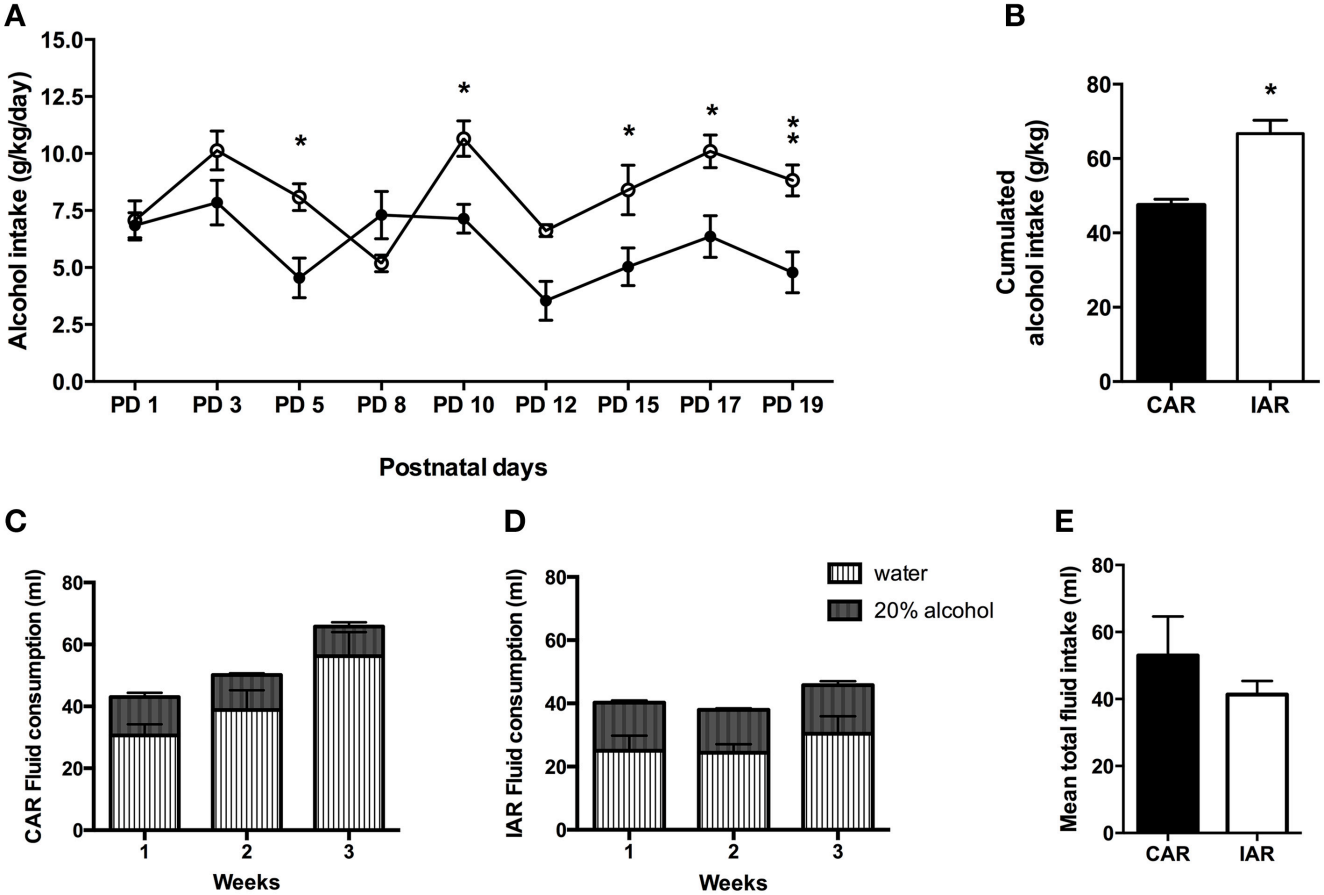
**Post-gestational drinking behavior**. **(A)** Alcohol intake (expressed in g/kg/day) during post-gestational days over 9 drinking sessions in female rats exposed to the home cage, 2-bottle “alcohol vs. water” choice regimen, under the following conditions: CAR, 20% v/v alcohol with continuous access; IAR-20% v/v alcohol with intermittent access. Bonferroni *post-hoc* test indicated that IAR significantly increased their alcohol intake with respect to CAR on PND5 (*t* = 3.161, *p* < 0.05), PND10 (*t* = 3.141, *p* < 0.05), PND15 (*t* = 3.005, *p* < 0.05), PND17 (*t* = 3.336, *p* < 0.05), and PND19 (*t* = 3.602, *p* < 0.01). **(B)** Total sum of alcohol consumed over the entire period. **(C)** Total fluid intake (ml) in CAR over the 3 post-gestational weeks. **(D)** Total fluid intake (ml) in IAR over the 3 post-gestational weeks. **(E)** Mean fluid consumption over the entire period. Each point or bar is the mean ± S. E. M. of *n* = 8 rats. ^*^p < 0.05, ^**^p < 0.01 vs. CAR.

Alcohol daily intake in CAR and IAR during lactation is shown in Figure 4A. The results of a RM Two-way ANOVA performed on g/kg/day of alcohol consumed during lactation revealed significant effect of lactation [*F*_(8, 112)_ = 6.65, *p* < 0.001], alcohol drinking pattern [*F*_(1, 14)_ = 17.22, *p* = 0.0010], and their interaction [*F*_(8, 112)_ = 4.07, *p* = 0.0003]. In particular, Bonferroni *post-hoc* test indicated, that IAR significantly increased their alcohol intake with respect to CAR (Figure 4A). A two tailed Student' *t*-test performed on the total amount of alcohol consumed over the 9 drinking sessions during lactation, showed that IAR significantly increased their amount of alcohol ingestion (*t* = 4.867, *df* = 14, *p* < 0.001), when compared to CAR (Figure 4B). Total fluid intake over the 3 lactation weeks is reported in Figure 4C (CAR) and Figure 4D (IAR).

Two-tailed Student's *t*-test on mean fluid intake over the 3 lactation weeks, indicated no significant differences between the two groups (*t* = 1.638, *df* = 4, *p* = 0.1768; Figure 4E).

**Figure 5:**
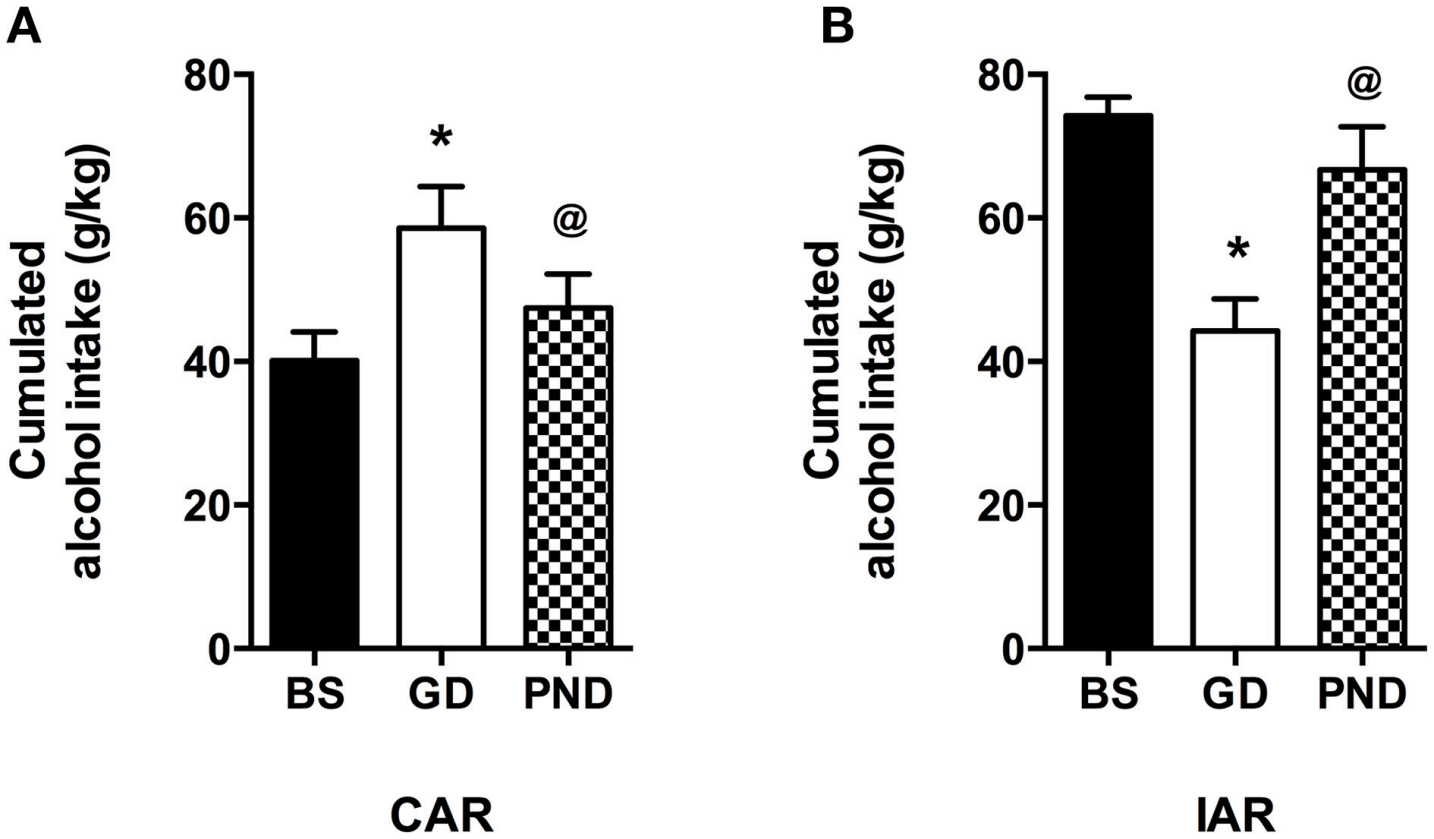
**Differences in total sum of alcohol intake (expressed in g/kg) between maternal alcohol consumption during gestation, lactation and baseline (last 3 weeks) intake in CAR (A) and IAR (B)**. Each value represents the mean ± S.D. of *n* = 8 rats. ^@^*p* < 0.05, ^*^p < 0.001 vs. baseline.

Alcohol drinking trajectories were also analyzed within CAR and IAR comparing maternal alcohol consumption during gestation and lactation to the respective pre-gestational basal intake during the last 9 pre-gestational drinking sessions. A one-way ANOVA for paired measures performed on CAR cumulative alcohol intake displayed a statistically significant effect [*F*_(1377)_ = 28.57, *p* < 0.0001]. Bonferroni *post-hoc* analysis showed that during gestation alcohol intake was increased with respect to basal intake (*t* = 7.508, *df* = 2, *p* < 0.001) while, during lactation it returned to the basal levels (*t* = 4.518, *df* = 2, *p* < 0.001; Figure 5A). When the analysis was performed on IAR drinking habit, the results obtained by a one-way ANOVA for paired measures showed a statistically significant effect [*F*_(3882)_ = 46.10, *p* < 0.0001]. Bonferroni *post-hoc* analysis indicated that during gestation IAR consumed a lower amount of alcohol with respect to the basal value while during lactation alcohol intake increased reaching the same levels as in pre-gestational time (*t* = 9.229, *df* = 2, *p* < 0.001; *t* = 6.911, *df* = 2, *p* < 0.001; Figure 5B).

#### Maternal behavior assessment

**Figure 6:**
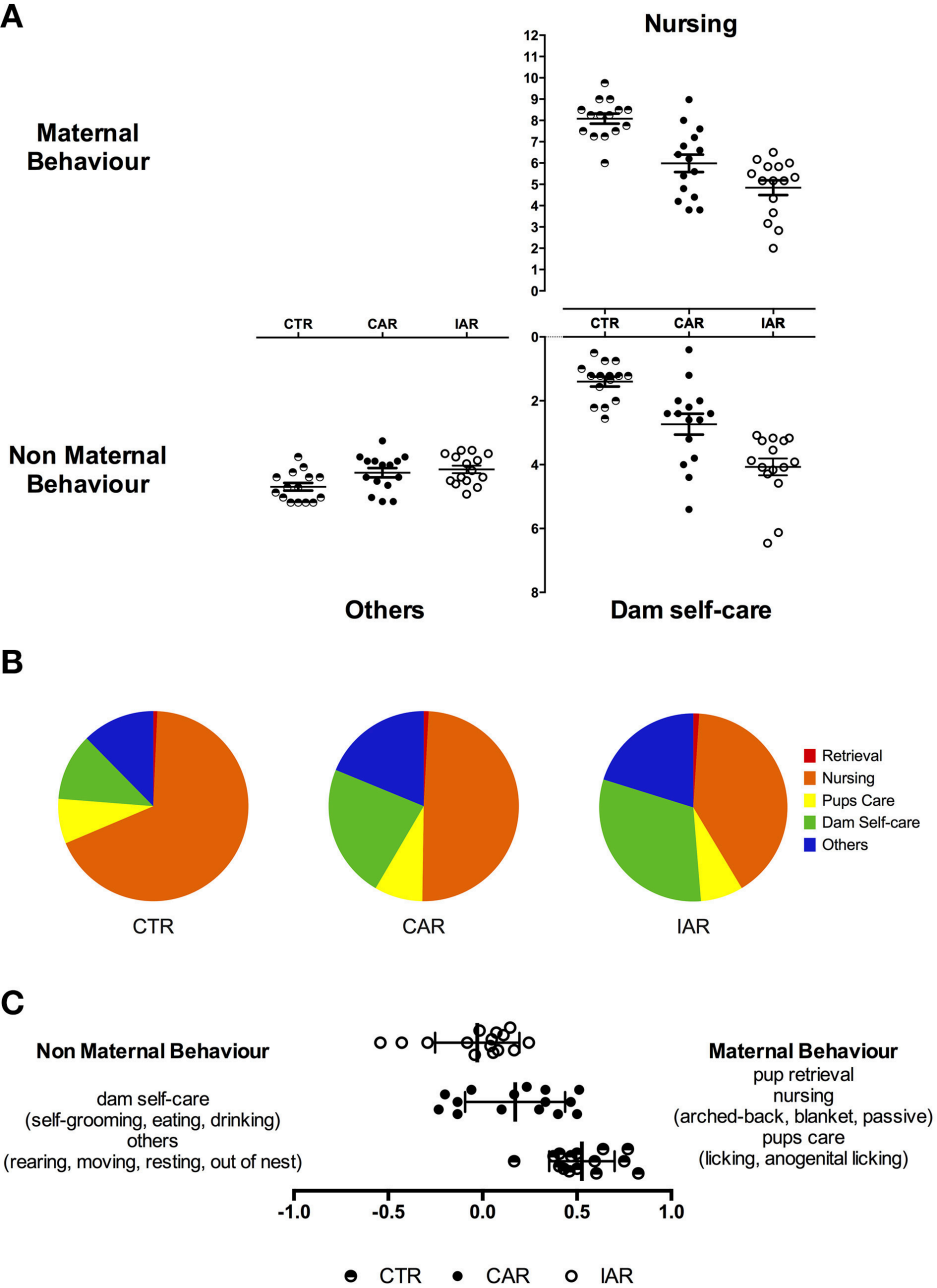
**Maternal behavior in female rats exposed to the home cage, 2-bottle “alcohol vs. water” choice regimen, under the following conditions: CAR, 20% v/v alcohol with continuous access; IAR-20% v/v alcohol with intermittent access**. **(A)** Evaluation of daily nursing (i. e., arched-back, blanket, passive nursing), dam self-care (i. e., self-grooming, eating, drinking), and other behavior scores (i. e., rearing, moving, resting, and standing out of nest). **(B)** Relative frequency of the maternal and non-maternal behavioral categories observed. **(C)** Maternal Behavior Index (MB-I). See text for details and significant differences.

Dam phenotypes from the different maternal and non-maternal categories were recorded 4 times a day, until PND21. Specific behaviors were scored and analyzed: retrieval, nursing, pup care, dam self-care, and “other behaviors.” Nursing, in terms of arched-back-, blanket- and passive nursing, was the most observed behavior The rank of the overall frequency of the behavioral categories over the 3 weeks of lactation resulted as follows (Figures 6A,B):

- CTR: nursing (67.92%) > others (12.36%) > dam self-care (11.39%) > pups care (7.64%) > retrieval (0.69%);
- CAR: nursing (49.39%) > dam self-care (22.78%) > others (18.78%) > pups care (8.20%) > retrieval (0.85%);
- IAR: nursing (40.37%) > dam self-care (31.11%) > others (20.19%) > pups care (7.31%) > retrieval (1.02%).

RM Two-way ANOVA was performed on daily nursing scores, with time as the within subject factor, and treatment as the between subject factor. The analysis showed significant effects of time [*F*_(14, 294)_ = 7.841, *p* < 0.001], treatment [*F*_(2, 21)_ = 52.15, *p* < 0.001], and their interaction [*F*_(28, 294)_ = 3.917, *p* < 0.001]. Results from Bonferroni *post-hoc* test indicated a significant reduction in nursing score in CAR and IAR with respect to CTR, and in IAR with respect to CAR (Table 1).

**Table 1:** **Evaluation of daily nursing score (i. e., arched-back, blanket, passive nursing) in female rats exposed to the home cage, 2-bottle “alcohol vs. water” choice regimen, under the following conditions: CAR-20% v/v alcohol with continuous access; IAR-20% v/v alcohol with intermittent access**.

**CAR vs. CTR**	**IAR vs. CTR**	**IAR vs. CAR**
**NURSING**		
PND 1 *t* = 2.829, *p* = 0.0149	PND 1 *t* = 6.287, *p* < 0.001	PND 1 *t* = 3.458, *p* = 0.0019
PND 2 *t* = 3.595, *p* = 0.0011	PND 3 *t* = 4.126, *p* = 0.0001	PND 10 *t* = 4.489, *p* < 0.001
PND 3 *t* = 3.301, *p* = 0.0032	PND 4 *t* = 3.635, *p* = 0.001	PND 17 *t* = 4.205, *p* < 0.001
PND 5 *t* = 3.654, *p* = 0.0009	PND 5 *t* = 4.912, *p* < 0.001	
PND 8 *t* = 2.535, *p* = 0.0352	PND 8 *t* = 3.831, *p* = 0.0005	
PND 12 *t* = 5.246, *p* < 0.001	PND 10 *t* = 2.751, *p* = 0.0198	
PND 15 *t* = 3.419, *p* = 0.0021	PND 11 *t* = 3.635, *p* = 0.001	
PND 16 *t* = 4.067, *p* = 0.0002	PND 12 *t* = 2.849, *p* = 0.014	
PND 19 *t* = 4.362, *p* < 0.001	PND 15 *t* = 3.144, *p* = 0.0055	
	PND 16 *t* = 6.189, *p* < 0.001	
	PND 17 *t* = 5.501, *p* < 0.001	
	PND 19 *t* = 6.287, *p* < 0.001	

RM Two-way ANOVA on dam self-care pattern showed that time [*F*_(14, 294)_ = 16.11, *p* < 0.001], treatment [*F*_(2, 21)_ = 61.40, *p* < 0.001] and their interaction [*F*_(28, 294)_ = 5.302, *p* < 0.001] were statistically significant. Bonferroni *post-hoc* test revealed a significant increase in dam self-care score in CAR and IAR with respect to CTR, and in IAR when compared with CAR (Table 2).

**Table 2:** **Evaluation of daily dam self-care score (i. e., self-grooming, eating, drinking) in female rats exposed to the home cage, 2-bottle “alcohol vs. water” choice regimen, under the following conditions: CAR-20% v/v alcohol with continuous access; IAR-20% v/v alcohol with intermittent access**.

**CAR vs. CTR**	**IAR vs. CTR**	**IAR vs. CAR**
**DAM SELF-CARE**		
PND 4 *t* = 2.488, *p* = 0.0401	PND 1 *t* = 4.561, *p* < 0.001	PND 5 *t* = 2.654, *p* = 0.0251
PND 12 *t* = 4.644, *p* < 0.001	PND 4 *t* = 3.317, *p* = 0.003	PND 10 *t* = 5.142, *p* < 0.001
PND 16 *t* = 4.810, *p* < 0.001	PND 5 *t* = 4.561, *p* < 0.001	PND 11 *t* = 4.091, *p* = 0.0002
PND 18 *t* = 3.981, *p* = 0.0003	PND 8 *t* = 2.903, *p* = 0.0119	PND 16 *t* = 3.483, *p* = 0.0017
PND 19 *t* = 5.391, *p* < 0.001	PND 10 *t* = 3.732 *p* = 0.0007	PND 17 *t* = 6.303, *p* < 0.001
	PND 11 *t* = 4.009, *p* = 0.0002	
	PND 15 *t* = 4.561, *p* < 0.001	
	PND 16 *t* = 8.294, *p* < 0.001	
	PND 17 *t* = 7.879, *p* < 0.001	
	PND 18 *p* = 0.0181, *t* = 2.765	
	PND PND19 *t* = 6.497, *p* < 0.001	

RM Two-way ANOVA on “other behaviors” showed that time [*F*_(14, 294)_ = 5.846, *p* < 0.001], treatment [*F*_(2, 21)_ = 8.030, *p* = 0.0026], and their interaction [*F*_(28, 294)_ = 1.584, *p* = 0.0340] were statistically significant. Bonferroni *post-hoc* test revealed a significant increase in dam self-care score in CAR and IAR with respect to CTR, and in IAR when compared with CAR (Table 3). RM two-way ANOVA on scores from retrieval and pup care (i.e., licking, anogenital licking) scores did not showed significant differences among the three experimental groups (Figures 6A,B).

**Table 3:** **Evaluation of daily “other behaviors” score (i. e., self-grooming, eating, drinking) in female rats exposed to the home cage, 2-bottle “alcohol vs. water” choice regimen, under the following conditions: CAR-20% v/v alcohol with continuous access; IAR-20% v/v alcohol with intermittent access**.

**CAR vs. CTR**	**IAR vs. CTR**	**IAR vs. CAR**
**OTHER BEHAVIORS**		
PND 5 *t* = 2.022, *p* = 0.0474	PND 3 *t* = 3.159, *p* = 0.0052	PND 9 *t* = 2.831, *p* = 0.0148
	PND 4 *t* = 3.286, *p* = 0.0034	
	PND 9 *t* = 2.907, *p* = 0.0117	

In order to obtain an overall snap-shot of the influence of the drinking patterns on dams' behavior, RM one-way ANOVA was carried out on MB-I data. The statistical analysis revealed a main effect of treatment on MB-I along the lactation period [*F*_(1907, 26.69)_ = 40.71, *p* < 0.001], with a significant reduction in CAR and IAR with respect to CTR (*t* = 5.818, *p* < 0.001; *t* = 9.751, *p* < 0.001), and in IAR when compared to CAR (*t* = 2.935, *p* < 0.05). The daily index of overall maternal behavior for each experimental group is shown in Figure 6C.

## Discussion

The present study aimed at evaluating pre-gestational-, gestational- and post-partum alcohol drinking trajectories in female rats employing a continuous- and an intermittent free-choice paradigm. The consequences of the long-term alcohol intake on different categories of reward processing, including preference for natural rewards and maternal behavior were analyzed, in order to provide an overall snapshot of the effects of alcohol free-consumption during discrete stages of female life and on such an important determinant as maternal care. The rationale of this study derives from recent reports showing that young women often continue their usual pattern of alcohol consumption into the early weeks of an unplanned pregnancy, and afterwards, thus exposing the fetus to alcohol teratogenity. Our results show that the continuous- and intermittent alcohol drinking paradigms were associated to differences in the drinking trajectories of female rats, both in pre-gestational time and during pregnancy or lactation; moreover, long-lasting alcohol intake affected discrete categories of maternal care in a pattern-related manner. The first evidence of this study regards the occurrence of an escalation in alcohol intake in female virgin rats exposed to the intermittent alcohol access protocol, from 4.5 g/kg/24 h in the first week to 8–9 g/kg/24 h afterwards. The daily amount of alcohol consumed did not display significant variations along the 12 weeks of the pre-gestational drinking sessions, and was significantly higher than that consumed by rats exposed to the continuous pattern of access. This result is consistent with other reports on male rats (Wise, 1973; Stuber et al., 2008; Hopf et al., 2010; Loi et al., 2010; Carnicella et al., 2014; Peris et al., 2015) and on Sardinian alcohol preferring female rats (Loi et al., 2014), that exhibited the same amount of consumption both immediately after the deprivation cycles and at the end of the 24-h drinking cycles, and displayed intoxicating blood alcohol levels (Loi et al., 2014), thus confirming that the intermittent pattern of alcohol consumption can reliably model alcohol abuse and binge-like drinking behavior (Crabbe et al., 2011). The observed escalation in alcohol intake can be correlated to loss of control over drinking and seems to be associated to the enhancement in the reinforcing and activating effects of alcohol on the mesolimbic system (Stuber et al., 2008). Accordingly, the saccharin preference test highlighted a greater reduction in sensitivity to natural reward in IAR than in CAR indicating that, although both alcohol-drinking patterns diverted motivational resources in female rats, a prominent drop in interest- or pleasure- into naturally rewarding stimuli occurred in IAR, in favor of a higher alcohol-related salience (Robinson and Berridge, 1993). The reduction in reward sensitivity is considered a core symptom of depression, although recent evidence highlights that the alteration of affective processing is not always associated with depressive symptoms in human alcoholics (Padula et al., 2015). Accordingly, continuous and intermittent alcohol exposures produce opposite effects in preclinical tests of depressive-like behavior, generating respectively increased immobility in the 1-day forced swim test in rats, and increased swimming in the re-exposure session of the Porsolt-test in mice (Getachew et al., 2010; Lee et al., 2015). Since a high prevalence of co-morbidity of affective disorders with alcoholism is reported in female human population (Wilsnack et al., 2013), a further characterization of the behavioral phenotype of long-lasting alcohol drinking female rats is mandatory to draw any conclusion on the occurrence of a depressive-like state.

Notably, after fecundation, alcohol drinking trajectories were evidently modified in that continuous free-access group increased its daily alcohol intake, while intermittent free-access rats decreased their daily consumption. These data are in agreement with previous findings on general female population showing that the reproductive states are able to modulate alcohol intake and preference (Little et al., 1976).

The decrease in gestational alcohol consumption recorded in IAR has also been observed in pregnant rodents, hamsters and monkeys exposed to alcohol (Carver et al., 1953; Elton and Wilson, 1977; Randall et al., 1980; Forger and Morin, 1982; Means and Goy, 1982) suggesting that intermittent alcohol access allows the onset of maternal adaptive and protective mechanisms for the fetus.

Opposite to IAR, CAR displayed an enhancement in their alcohol intake with respect to the baseline levels: in these animals chronic continuous alcohol access might be responsible for modifications of receptor sensitivity and behavioral tolerance, that sustained and promoted alcohol drinking during pregnancy.

Post-partum days are characterized by physiological reduction in the dopaminergic tone, as lactation begins, and notably, alcohol intake in IAR was increased to baseline levels, reproducing the same trajectories as in the pre-gestational period. Also CAR drinking levels returned to the basal values. Again, the physiological responses of rodents to alcohol are similar to the human ones (Driscoll et al., 1990; Hannigan, 1996) and the neuro-behavioral outcomes of perinatal alcohol exposure have been fairly consistent with clinical and behavioral outcomes in human studies showing that a significant proportion of new mothers resume alcohol consumption and even engage in binge drinking within a year post-delivery (Jagodzinski and Fleming, 2007; Muhuri and Gfroerer, 2009; Laborde and Mair, 2012). Thus, findings from animal studies on drinking behavior in perinatal time may lead to speculate on the behavior of women who drink during pregnancy and lactation. Psychophysical, “economical” and logistical challenges due to pregnancy and child birth mark an important transition in a woman's life (Rutter, 1996), and may either play a protective role on drinking or lead to a stress-related increase in alcohol intake that interferes with an appropriate infant rearing environment (Jester et al., 2000; Wolfe, 2009; Fergusson et al., 2012; Staff et al., 2014). In clinical studies, analysis of the specific effects of alcohol consumption during pregnancy on maternal care is complicated, due to confounding factors, such as pattern, dose, and timing of alcohol consumption; poly-drug use; maternal age; maternal mental health history; and socioeconomic status (Olson et al., 2001; Knudsen et al., 2015). Conversely, maternal behavior has been studied intensively in the animal model and in the rat in particular (Numan and Sheehan, 1997; Fleming et al., 1999, 2002), contributing significantly to the exploration of this field of research. Maternal care in the rat is the result of a complex interplay of the hormonal milieu of the dam and the behavior of both pup and dam. Disruption of the maternal-pup dyad is known to have critical impact on the long-term outcome of the pups (Meaney, 2001; Champagne and Meaney, 2007). Unfortunately, studies on the consequences of alcohol consumption during pregnancy on maternal care in the animal model have reported inconsistent results. In some studies alcohol intake during pregnancy did not alter maternal behavior (Ewart and Cutler, 1979; Anandam et al., 1980), in some others pup retrieval was delayed or reduced (Abel, 1978; Ness and Franchina, 1990), and the combined exposure to alcohol and nicotine produced increased time away from pups (McMurray et al., 2008).

Evidence from this study shows that in both alcohol-drinking groups long-term alcohol consumption yielded significant disruptions in nursing and overall maternal response, increasing the frequency of non-pup directed behaviors, such as dam self-care and other behaviors throughout the postpartum period. Interestingly, IAR showed greater reduction in frequency of nursing, and time spent in the nest with the offspring than CAR. At the same time, IAR showed higher frequency of non-maternal behaviors, with a significant increase in dam self-care. The increase in dam-self care indicates that not all the behavioral categories of affectivity were impaired. Self-grooming is an ethologically relevant behavior associated with affective state in rodents. The grooming analysis allows to infer about self-care and motivational behavior, since sleep deprivation in rat dams, as well as chronic stress in mice, decreased number and duration of grooming episodes (Santarelli et al., 2003; Pires et al., 2012). However, dam self-care included drinking behavior, whose increase could reflect higher salience attributed to alcohol than to the natural source of reinforcement, represented by the pups, after parturition (Fleming et al., 1994; Lee et al., 1999).

Notably, alcohol consumption in the free-access paradigms during pregnancy and lactation did not change the frequency of retrieval, pup licking and grooming compared with that of naive dams, as reported in other studies (Pepino et al., 2002; Pueta et al., 2008). Deficits in maternal behavior of alcohol-treated dams could manifest from changes in pups' behavior toward the mother. Rat pups prenatally exposed to alcohol have a longer latency to nipple attach and reduced ultrasonic vocalizations (Chen et al., 1982; Rockwood and Riley, 1990; Kehoe and Shoemaker, 1991), which could limit alcohol-exposed pups' ability to elicit the same levels of maternal care as controls. This is further supported by cross-fostering experiments in which control mothers also reduced maternal behavior when caring for alcohol-treated pups (Subramanian, 1992) even if cross-fostering itself can disrupt behavior so that results and interpretation from those studies require extensive attention. Maternal behavior is also disrupted in alcohol exposed female rats that display deficits in retrieval and caring naive pups (Wilson et al., 1996). As a matter of fact, in the current study, we were not interested at splitting alcohol effect on the mother from alcohol effect on the offspring, since in the human condition cross fostering is not applicable. Rather, the aim of this research was to make a longitudinal evaluation of the consequences of different patterns of alcohol consumption on the mother-infant dyad, whose integrity is an absolute requirement for the full development of stress responsiveness, adult parenting and social behavior of the exposed offspring.

Neural circuits associated with parenting significantly overlap with those involved in alcohol abuse (i.e., frontal, striatal, and limbic systems; Insel, 2003; Zhou et al., 2006). The dopaminergic neurotransmission plays a role in specific aspects of maternal behavior as well as in reward (Byrnes et al., 2002): dopamine can activate oxytocin release into VTA and nucleus accumbens thus contributing to the rewarding value of pup stimuli (Lee et al., 1999). Interfering with dopaminergic- and oxytocinergic activity in these regions results in decreasing salience attribution toward the offspring. Therefore, the evidence from this study prompts us to hypothesize, that long-lasting alcohol intake during pre-gestational time, pregnancy, and lactation is able to reduce nursing, and increase self-directed behaviors, in a pattern related manner, as a result of a disarrangement of the neuronal and hormonal milieu that underlie maternal behavior and incentive salience attribution. As far as we know, this is the first study that reports extensive observations on maternal behavior in dams that had long term free-access to alcohol during pre-gestational time, pregnancy, and lactation in the home cage setting, without disturbing either the mother or the offspring. Our maternal behavioral data corroborate the very few clinical studies that focus on human maternal care and meet the need for modeling human alcohol habit and its consequences on the mother-infant dyad searching for the molecular and cellular substrates underlying the behavioral phenotypes. Clinical prevention and treatment guidelines should tackle gestational, and perinatal alcohol consumption but also excessive alcohol intake however it occurs, either continuously or as binge drinking episodes, especially in young women at fertile age.

## Author contributions

AB: experimental procedures; contribution to experimental design and writing. FP: statistical analysis and graphical layout; contribution to writing. AC: experimental procedures. GL: experimental procedures. CC: experimental design and writing.

## Funding

Supported by PO.FESR 2007/2013.

### Conflict of interest statement

The authors declare that the research was conducted in the absence of any commercial or financial relationships that could be construed as a potential conflict of interest.
